# Gallbladder Cancer: Current Insights in Genetic Alterations and Their Possible Therapeutic Implications

**DOI:** 10.3390/cancers13215257

**Published:** 2021-10-20

**Authors:** Hendrien Kuipers, Tessa J. J. de Bitter, Marieke T. de Boer, Rachel S. van der Post, Maarten W. Nijkamp, Philip R. de Reuver, Rudolf S. N. Fehrmann, Frederik J. H. Hoogwater

**Affiliations:** 1 Department of Surgery, Section Hepato-Pancreato-Biliary Surgery and Liver Transplantation, University of Groningen, University Medical Center Groningen, Hanzeplein 1, 9713 GZ Groningen, The Netherlands; m.t.de.boer@umcg.nl (M.T.d.B.); m.w.nijkamp@umcg.nl (M.W.N.); 2 Department of Pathology, Radboud University Medical Center, Geert Grooteplein Zuid 10, 6525 GA Nijmegen, The Netherlands; tessa.jj.debitter@radboudumc.nl (T.J.J.d.B.); chella.vanderpost@radboudumc.nl (R.S.v.d.P.); 3 Department of Surgery, Radboud University Medical Center, Geert Grooteplein Zuid 10, 6525 GA Nijmegen, The Netherlands; philip.dereuver@radboudumc.nl; 4 Department of Medical Oncology, University of Groningen, University Medical Center Groningen, Hanzeplein 1, 9713 GZ Groningen, The Netherlands; r.s.n.fehrmann@umcg.nl

**Keywords:** gallbladder cancer, gene mutations, genetic alterations, tumor mutational burden, microsatellite instability, targeted therapy

## Abstract

**Simple Summary:**

Knowledge of genetic alterations in gallbladder cancer (GBC) continues to increase. This systematic review provides an overview of frequently occurring genetic alterations in GBC and describes their possible therapeutic implications. We detected three frequently (>5%) altered genes (*ATM*, *ERBB2* and *PIK3CA*) for which targeted therapies are available in other cancer types. For solid cancers with microsatellite instability or a high tumor mutational burden pembrolizumab is FDA-approved. Altogether, these five biomarkers might be used in future molecular panels to enable precision medicine for patients with GBC. We found only nine clinical trials evaluating targeted therapies in GBC directed at frequently altered genes (*ERBB2*, *ARID1A*, *ATM* and *KRAS*). This underlines the challenges to perform such clinical trials in this rare, heterogeneous cancer type and emphasizes the need for multicenter clinical trials.

**Abstract:**

Due to the fast progression in molecular technologies such as next-generation sequencing, knowledge of genetic alterations in gallbladder cancer (GBC) increases. This systematic review provides an overview of frequently occurring genetic alterations occurring in GBC and their possible therapeutic implications. A literature search was performed utilizing PubMed, EMBASE, Cochrane Library, and Web of Science. Only studies reporting genetic alterations in human GBC were included. In total, data were extracted from 62 articles, describing a total of 3893 GBC samples. Frequently detected genetic alterations (>5% in >5 samples across all studies) in GBC for which targeted therapies are available in other cancer types included mutations in *ATM*, *ERBB2*, and *PIK3CA*, and *ERBB2* amplifications. High tumor mutational burden (TMB-H) and microsatellite instability (MSI-H) were infrequently observed in GBC (1.7% and 3.5%, respectively). For solid cancers with TMB-H or MSI-H pembrolizumab is FDA-approved and shows an objective response rates of 50% for TMB-H GBC and 41% for MSI-H biliary tract cancer. Only nine clinical trials evaluated targeted therapies in GBC directed at frequently altered genes (*ERBB2*, *ARID1A*, *ATM*, and *KRAS*). This underlines the challenges to perform such clinical trials in this rare, heterogeneous cancer type and emphasizes the need for multicenter clinical trials.

## 1. Introduction

Gallbladder cancer (GBC) is a relatively uncommon malignancy, with a worldwide incidence of less than 2 per 100,000 [1]. However, GBC shows a broad geographical and ethnic distribution, with low incidence rates in developed countries and higher incidence rates in South American countries, India, Pakistan, Japan and Korea. Higher incidence rates are also observed among Mexican Americans, Indian Americas, and Eastern Europeans [1,2,3]. Well-established risk factors include age, obesity, female gender, family history, cholelithiasis, and anomalous junction of the pancreatobiliary duct [4,5,6].

At present, radical resection by cholecystectomy with lymphadenectomy of the hepatoduodenal ligament and wedge or segment resection of the liver is the only treatment with curative intent [7]. Unfortunately, most patients present at an advanced stage and are unresectable [8,9]. Even after resection, the five-year survival rate ranges from 18% to 34% [10,11]. Palliative systemic chemotherapy for patients with GBC has shown limited efficacy. In patients with locally advanced or metastatic biliary tract cancer (BTC), including GBC, the ABC-02 trial reported a median overall survival of 11.7 months in the BTC group treated with gemcitabine plus cisplatin and 8.1 months in the BTC group treated with gemcitabine monotherapy [12].

With the rapid developments in next-generation sequencing (NGS), our knowledge of genetic alterations occurring in GBC has increased over the past decade [13]. Consequently, the number of preclinical studies and clinical trials evaluating therapies targeting these genetic alterations is slowly increasing [14]. Several reviews on genetic alterations in biliary tract cancer have been published. However, a systematic review specifically focusing on genetic alterations in GBC and their therapeutic implications is lacking.

## 2. Materials and Methods

### 2.1. Literature Search

The protocol for this systematic review was prospectively registered in the PROSPERO registry (CRD42021265246). The study was conducted according to the Preferred Reporting Items for Systematic Reviews and Meta-Analyses (PRISMA) guidelines [15].

A literature search was performed until 26 March 2021 in PubMed, EMBASE, Cochrane Library, and Web of Science. Keywords or medical subject headings (MeSH) used for the search are provided in Appendix Table A1. Only full-text articles published in English in 2000 or afterwards were selected.

### 2.2. Study Selection

Citations were deduplicated by using tools in Endnote and Rayyan (rayyan.ai). Subsequently, all duplications were manually verified. Titles and abstracts of all retrieved records were then independently screened for eligibility by two investigators (H.K. and T.J.J.d.B.) using the Rayyan platform for systematic reviews. Discordance was re-evaluated by both investigators and resolved by consensus after discussion. Full papers were obtained for records that were considered potentially eligible by both investigators. In the case of studies with overlapping data, the article reporting the largest cohort was selected.

Only studies were included that identified somatic genetic alterations in human GBC with Polymerase Chain Reaction (PCR) with Sanger sequencing, NGS (targeted, whole-exome, and whole-genome sequencing), MassArray, or SNaPshot. Studies using immunohistochemistry or FISH techniques were excluded. Case reports, retracted articles, and preliminary results were excluded, as well as studies focusing on gene expression, genetic alterations in cell lines, cell-free DNA from serum, and mitochondrial DNA.

This review focused on adenocarcinoma of the gallbladder. Other histologies, i.e., adenosquamous, squamous, neuroendocrine, and sarcomatoid carcinoma, were excluded. In case no histological type was reported, tumors were assumed to represent adenocarcinomas since only 5% of GBC have histology other than adenocarcinoma [16,17].

### 2.3. Data Extraction

Data extracted included first author, year of publication, study population, sequencing technique, number of samples, and reported genetic alterations and their frequencies. Included alterations were non-synonymous mutations and copy number aberrations (DNA amplifications and deletions). In addition, frequencies of high tumor mutational burden (TMB) and the presence of microsatellite instability (MSI) were extracted.

Only frequently occurring genetic alterations (>5% [18,19] across all studies and in >5 of all included GBC samples) were included. Per genetic alteration, weighted average of reported frequencies was calculated by using the number of samples analyzed in a study as the weight.

A column scatter plot was constructed for all frequently occurring genetic alterations. Bars displayed the minimum and maximum reported frequency. Dots represented the frequency per study. Diamonds represented the weighted averages.

No risk of bias was assessed since no methods are available that could assess confounders such as inter-population diversity and variations in DNA techniques [20,21]. Nevertheless, to facilitate the reader to assess the studies’ quality and risk of bias, details of each included study were displayed in tables.

### 2.4. Therapeutic Implications

Actionable alterations were identified by comparing the frequently altered genes detected in this study with the actionable genes of the OncoKB database (accessed 21 July 2021) [22]. Only actionable genes with level 1 and 2 therapeutic evidence in solid tumors were analyzed. Level 1 evidence was defined as a U.S. Food and Drug Administration (FDA)-recognized biomarker predictive of response to FDA-approved drugs for a specific indication and level 2 as a standard care biomarker predictive of response to FDA-approved drugs for another indication. Data extracted included targetable alteration, drug, level of evidence, and cancer type.

Second, clinicaltrials.gov and clinicaltrialsregister.eu were examined for clinical trials targeting frequently occurring genetic alterations detected in this study in patients with GBC or BTC (accessed 26 July 2021).

## 3. Results

### 3.1. Literature Search and Study Selection

**Figure 1:**
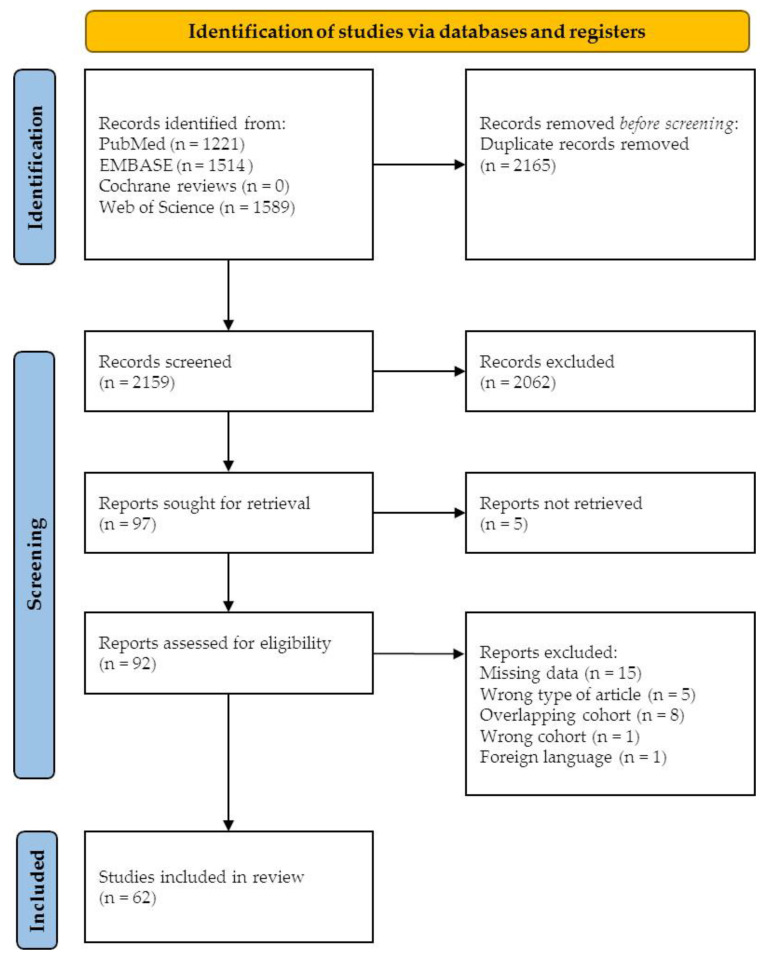
Identification of studies via databases.

In total, 4324 records were retrieved from all databases. After removing duplicate records, 2159 records were screened for eligibility based on title and abstract (Figure 1). A total of 92 articles underwent full-text reading. Out of 92 articles, 30 were excluded due to: no data for adenocarcinoma of the gallbladder available (N = 12), overlapping cohorts (N = 8), the wrong type of article (N = 5), no genetic alteration frequency data available (N = 3), wrong study population (N = 1), and not written in English (N = 1). A total of 62 articles were included for final data extraction, describing a total of 3893 GBC samples from individual patients [18,19,23,24,25,26,27,28,29,30,31,32,33,34,35,36,37,38,39,40,41,42,43,44,45,46,47,48,49,50,51,52,53,54,55,56,57,58,59,60,61,62,63,64,65,66,67,68,69,70,71,72,73,74,75,76,77,78,79,80,81,82].

### 3.2. Most Frequently Mutated Genes

**Figure 2:**
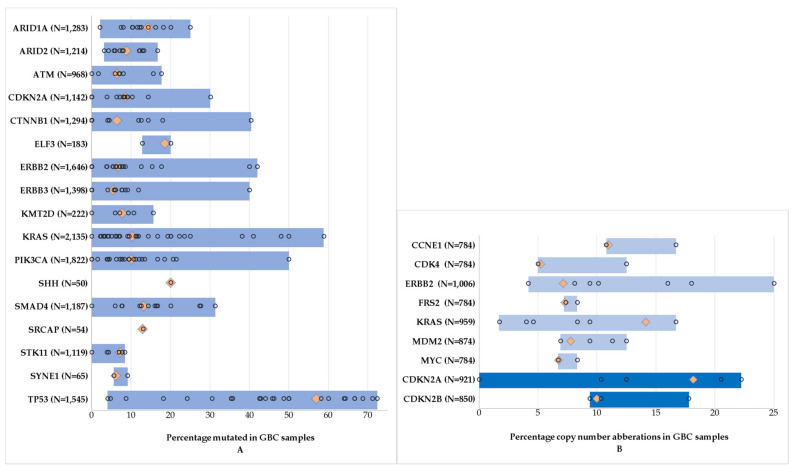
Frequently occurring genetic alterations (>5% across all studies and in >5 of all included samples) in gallbladder cancer. Numbers represent the total number of samples tested for this gene. Blue bars represent the minimum and maximum reported alteration frequencies. Dots represent the mutation frequency per study. Orange diamonds represent the weighted average of all frequencies. (**A**) Gene mutations. (**B**) Gene amplifications (light blue) and gene deletions (dark blue).

Figure 2A represents all frequently occurring gene mutations in GBC. Appendix Table A2 shows the results for all frequent gene mutations. *TP53* was the most frequently mutated gene in GBC (weighted average 57%), followed by *SHH (*weighted average 20%), *ELF3 (*weighted average 19%), *ARID1A* (weighted average 14%), and *SMAD4 (*weighted average 13%).

### 3.3. Amplifications and Deletions

Ten studies reported frequencies of copy number aberrations occurring in GBC (Figure 2B). Frequently occurring copy number alterations included amplifications of the oncogenes *CCNE1*, *CDK4, ERBB2, FRS2*, *KRAS*, *MDM2,* and *MYC* (Table 1). Deletions were observed in *CDKN2A* and *CDKN2B*.

### 3.4. Tumor Mutational Burden (TMB)

The reported frequencies of TMB ranged between 2.6 and 7.03 mutations/Megabase (Mb) in GBC samples (Table 2). Among 864 patients with known TMB status, 15 patients (1.7%) had high TMB (TMB-H). However, the threshold to define TMB-H varied between studies.

### 3.5. Microsatellite Instability (MSI)

Table 3 outlines all studies (N = 13) reporting on MSI status in 1162 patients with GBC. This table shows that a high diversity of marker panels and MSI definitions were used. A broad range of MSI-high frequencies were reported, although most studies reported a low incidence. The weighted average for MSI-high frequency was 3.5%.

### 3.6. Possible Therapeutic Implications

#### 3.6.1. Targetable Alterations in Other Malignancies

Currently, no FDA-approved therapies targeting gene mutations or copy number aberrations are available for GBC. However, several targeted therapies directed at actionable alterations that were also frequently observed in GBC are FDA-approved in other cancers (Table 4). These alterations include mutations in *ATM*, *PIK3CA* and *ERBB2,* and amplifications in *ERBB2*. For solid cancers with MSI-H or TMB-H, pembrolizumab is approved by the FDA.

#### 3.6.2. Clinical Trials

Table 5 presents all clinical trials that include GBC patients harboring frequently occurring genetic alterations. No completed studies were identified; all studies are currently recruiting participants.

Most studies evaluate targeted therapies directed at *ERBB2* alterations and related signal pathway components. One trial assesses the safety and efficacy of ELI-002 immunotherapy, a novel amphiphile therapeutic vaccine targeting KRAS-driven cancers, for patients with *KRAS* or *NRAS* mutations (G12D or G12R) in various solid tumor types including GBC (NCT04853017). Moreover, a phase 2 trial analyzes the efficacy and toxicity of olaparib in patients with metastatic BTC with an DNA repair gene mutation, including *ARID1A* and *ATM* (NCT04042831). The NCT02091141 trial evaluates six treatment regimens based on molecular testing in patients with advanced solid tumors, including BTC. For example, patients with tumors demonstrating elevated TMB (≥10 mutations/Mb) will receive atezolizumab.

## 4. Discussion

In this systematic review of 62 articles assessing genetic alterations in GBC, 3893 GBC samples were analyzed. Frequently occurring genetic alterations included mutations in 17 genes and amplifications/deletions in nine genes. Since no targeted therapy is currently available for GBC, frequent genetic alterations detected in this study were matched to actionable genetic alterations in other solid tumors. Only mutations in *ATM*, *ERBB2* and *PIK3CA* and amplifications in *ERBB2* are currently targetable with FDA-approved drugs in other solid tumors. Therefore, these alterations are potential targets in GBC and might be included in future molecular testing panels for personalized treatment decisions.

In GBC, the prevalence of TMB-H was low. Previous studies have shown that TMB-H is correlated with clinical response to immunotherapy in several tumors, including BTC, and that patients with TMB-H tumors can benefit from immune checkpoint inhibitors such as pembrolizumab [108,109,110]. In patients with GBC receiving immunotherapy, a higher objective response rate was observed for TMB-high tumors compared to TMB-low tumors (50% vs. 25%) [47]. Pembrolizumab has been FDA-approved for patients with TMB-H advanced solid cancers.

Another biomarker associated with response to immune checkpoint inhibitors is MSI, which was observed in 3.5% of patients in the present study. For patients with MSI-H cancers, pembrolizumab is also approved by the FDA, as well as by the Pharmaceuticals and Medical Devices Agency (PMDA, Japan). The European Medicines Agency (EMA, European Union) and the National Medical Products Administration (NMPA, China) have not yet given approval for this agent in patients with MSI-H (or TMB-H). In patients with cholangiocarcinoma who received pembrolizumab, an objective response rate of 41% was seen [111]. Therefore, although only beneficial in a small proportion of patients, both TMB-H and MSI-H are interesting biomarkers in precision medicine for GBC.

A broad range was observed in genetic alteration frequencies in GBC across all studies. In part, this could be attributed to differences in sequencing technologies and assessed gene regions. For example, NGS has a slightly lower sensitivity for mutation detection compared to some other technologies like real-time PCR testing, although detecting low-frequency genetic alterations through (ultra-) deep sequencing with a higher coverage can partly overcome this issue [112,113,114]. Moreover, geographical differences in mutation patterns might be present, as previous studies have shown [35,46,73]. However, no one-to-one regional comparisons of genetic alteration frequencies could be made due to different sequencing techniques used throughout all studies. Substantial differences were also observed regarding the frequency of MSI. A large variation in marker panels and MSI definitions, and some missing MSI definitions, complicated comparison of all MSI frequencies. Although a weighted average of MSI frequency was calculated to minimalize the influence of sample size on the average frequency, our results should be taken with caution due to the heterogeneity in MSI panels and definitions. Usage of the National Cancer Institute (NCI) panel of microsatellites and definitions in future research would facilitate MSI comparison among studies [115].

Since some genetic alterations might impede the efficacy of therapies targeting other genetic alterations, co-existing alterations might be an important determinant for therapeutic sensitivity and resistance. For example, patients with solid stage IV cancer types harboring amplification in the *MDM2* family or *EGFR* aberrations who received anti-PD1/PDL1 immunotherapy showed increased tumor growth [116]. Unfortunately, co-existing alterations could not be assessed in this study due to differences in sequencing techniques and missing data in some studies.

Only nine clinical trials which evaluated targeted therapies directed at frequently occurring genetic alterations in GBC were identified. Though we assessed only trials targeting these genetic alterations specifically, these limited results underline that GBC remains a relatively under-investigated cancer type regarding therapeutic agents. Unfortunately, initiation of clinical trials including patients with GBC is logistically challenging since GBC is a rare cancer type in most Western countries. The large inter-tumor heterogeneity of GBC poses another challenge for targeted therapies. However, basket trials, a clinical trial that tests agents in different cancer types with the same genetic alteration, could provide a solution to this challenge in future studies.

This study has several limitations. First, histological types and subtypes of GBC samples could not always be retrieved. It is important to report histological (sub)types to be able to determine whether differences in genetic alterations are associated with different histology. Second, a publication bias might exist. Therefore, actual overall alteration frequencies might be over- or underestimated.

## 5. Conclusions

In conclusion, GBC has a diverse mutational landscape. Frequently occurring genetic alterations in GBC that are actionable in other solid tumors are rare, including mutations in *ATM*, *ERBB2*, *PIK3CA* and amplifications in *ERBB2.* For all solid tumors with MSI-high or TMB-high, including GBC, the immune checkpoint inhibitor pembrolizumab is FDA-approved. Few clinical trials targeting the frequently altered genes in GBC are performed, emphasizing the need for multicenter clinical basket trials.

## Figures and Tables

**Table 1:** Copy number aberrations in gallbladder cancer.

Gene	WA	N	Frequency	Methods	Histology	Population	Author	Year	Ref.
*CCNE1*	11.0%	82/760	11%	NGS	N.A.	America	Abdel-Wahab	2020	[19]
		4/24	17%	NGS	N.A.	America	Okamura	2021	[27]
*CDK4*	5.2%	38/760	5%	NGS	N.A.	America	Abdel-Wahab	2020	[19]
		3/24	13%	NGS	N.A.	America	Okamura	2021	[27]
*CDKN2A*	18.2%	156/760	21%	NGS	N.A.	America	Abdel-Wahab	2020	[19]
		6/58	10%	ultra-deep targeted NGS	AC	China	Lin	2019	[70]
		4/32	13%	targeted NGS	AC	Chile, Japan	Narayan	2019	[73]
		0/25	0%	targeted exome sequencing	N.A.	Korea	Chae	2019	[79]
		10/45	22%	real-time PCR	AC	Japan	Tadokoro	2007	[83]
*CDKN2B*	10.0%	135/760	18%	NGS	N.A.	America	Abdel-Wahab	2020	[19]
		6/58	10%	ultra-deep targeted NGS	AC	China	Lin	2019	[70]
		3/32	9%	targeted NGS	AC	Chile, Japan	Narayan	2019	[73]
*ERBB2*	7.1%	77/760	10%	NGS	N.A.	America	Abdel-Wahab	2020	[19]
		1/24	4%	NGS	N.A.	America	Okamura	2021	[27]
		1/4	25%	TS	AC	Korea	Yoo	2016	[37]
		9/111	8%	NGS	N.A.	America	Mondaca	2019	[38]
		9/50	18%	NGS	N.A.	India	Patel	2020	[39]
		3/32	9%	targeted NGS	AC	Chile, Japan	Narayan	2019	[73]
		4/25	16%	targeted exome sequencing	N.A.	Korea	Chae	2019	[79]
*FRS2*	7.3%	55/760	7%	NGS	N.A.	America	Abdel-Wahab	2020	[19]
		2/24	8%	NGS	N.A.	America	Okamura	2021	[27]
*KRAS*	14.1%	35/760	5%	NGS	N.A.	America	Abdel-Wahab	2020	[19]
		2/24	8%	NGS	N.A.	America	Okamura	2021	[27]
		10/60	17%	PCR + DS	AC	Taiwan	Huang	2017	[67]
		1/58	2%	ultra-deep targeted NGS	AC	China	Lin	2019	[70]
		3/32	9%	targeted NGS	AC	Chile, Japan	Narayan	2019	[73]
		1/25	4%	targeted exome sequencing	N.A.	Korea	Chae	2019	[79]
*MDM2*	7.8%	86/760	11%	NGS	N.A.	America	Abdel-Wahab	2020	[19]
		3/24	13%	NGS	N.A.	America	Okamura	2021	[27]
		4/58	7%	ultra-deep targeted NGS	AC	China	Lin	2019	[70]
		3/32	9%	targeted NGS	AC	Chile, Japan	Narayan	2019	[73]
*MYC*	6.8%	51/760	7%	NGS	N.A.	America	Abdel-Wahab	2020	[19]
		2/24	8%	NGS	N.A.	America	Okamura	2021	[27]

WA: weighted average (calculated by using the number of samples analyzed in a study as the weight); N.A.: not available; AC: adenocarcinoma; NGS: next-generation sequencing; PCR: polymerase chain reaction; DS: direct sequencing; TS: targeted sequencing.

**Table 2:** Tumor mutational burden in gallbladder cancer.

Author	Origin	Histology	N	TMB (Mut/Mb [Range])	TMB-H Definition	TMB-H	Ref.
Patel	India	N.A.	43	5 (1–14)	-	-	[39]
Weinberg	America	N.A	104	-	≥17 mut/Mb	6/104 (5.8%)	[59]
Li	China	N.A.	12	7.03	-	-	[47]
Abdel-Wahab	America	N.A.	760	2.6 (0–403)	≥19.5 mut/Mb	9/760 (1.2%)	[19]

TMB: tumor mutational burden; TMB-H: high tumor mutational burden; mut: mutations; Mb: megabase.

**Table 3:** Microsatellite instability in gallbladder cancer.

Author	Origin	Histology	MSI Markers	MSI Definition	MSI	Ref.
Nagai	Japan	N.A.	D2S97, D6S477, D8S339, D9S131, D10S197, D17S796, D18S36, TP53 (17p12), DCC (18q21), APC (5q21)	Shifts in ≥30% of markers	7/17 (41%)	[24]
Kim	Korea	N.A.	3p12-22 (D3S1274, D3S4103, D3S1766) 5q11-23 (D5S107, D5S409, IRF1) 8p22-23 (D8S254, D8S261) 9p22 (IFNA, D9S126, D9S104) 13q13-14 (D13S118 and D13S133) 17p11-13 (D17S786, D17S796,TP53) 18q12-21 (D18S34)	Shifts in ≥1 marker	3/15 (20%)	[31]
Nagahashi	Japan, Hungary	AC	NCI: BAT-25, BAT-26, D2S123, D5S346, D17S250	Shifts in ≥2 markers	9/34 (27%)	[35]
Patel	India	N.A.	Genome-wide analysis of 95 loci	N.A.	0/43 (0%)	[39]
Abdel-Wahab	U.S.	N.A.	114 loci	Shifts in ≥2 markers	3/551 (1%)	[19]
Wistuba	Chile	AC	81 loci on 3p, 8p, 9q and 22q	Shifts in ≥1 marker	6/12 (50%)	[43]
Pandey	Chile, Korea, India	AC	Exome-wide analysis	MSI score > 0.35	3/152 (2%)	[46]
Li	China	N.A.	NGS	N.A.	0/12 (0%)	[47]
Rashid	China	AC	NCI (BAT-25, BAT-26, D2S123, D5S346, D17S250) and TGFβRII	Shifts in ≥40% of D2S123, D5S346, D17S25, or alteration of BAT-25, BAT-26 or TGFβRII	2/64 (3%)	[49]
Goeppert	Germany	AC	BAT25, BAT26, and CAT25	Shifts in ≥2 markers	1/69 (1%)	[52]
Yoshida	Japan	AC	p53, APC, DCC, NM23-H1, D2S123, D3S1029, D5S107, D17S261, D18S34	Shifts in ≥33% of markers	0/30 (0%)	[54]
Roa	Chile	AC	NCI: BAT25, BAT26, D2S123, D5S346, D17S250 and BAT40, D3S1067, D3S1286, D3S1262, D3S1478, D12S1638, D12S347, D16S265	Shifts in >30% of markers	6/59 (10%)	[55]
Weinberg	U.S.	N.A.	Targeted NGS over 7000 loci	N.A.	1/104 (1%)	[59]

MSI: microsatellite instability; N.A.: not available; AC: adenocarcinoma; NCI: National Cancer Institute recommendation; NGS: next-generation sequencing.

**Table 4:** FDA-approved drugs targeting genetic alterations in other malignancies.

Target	Level	Malignancy	Agent	ORR	Ref.
*ATM* ^¥^	1	Prostate cancer	Olaparib	*BRCA1*, *BRCA2*, or *ATM:* 28/84 (33%)	[84]
*ERBB2* *	1	Esophagogastric cancer	Pembrolizumab + trastuzumab + chemotherapy	32/35 (91%)	[85]
			Trastuzumab + chemotherapy	139/294 (47%)	[86]
			Trastuzumab deruxtecan	61/119 (51%)	[87]
*ERBB2* *	1	Breast cancer	Ado-trastuzumab emtansine	173/397 (44%)	[88]
			Lapatinib + letrozole	31/111 (28%)	[89]
			Lapatinib + capecitabine	36/163 (22%)	[90]
			Margetuximab + chemotherapy	67/266 (25%)	[91]
			Neratinib	34/117 (29%)	[92]
			Trastuzumab	30/114 (26%)	[93]
			Trastuzumab + pertuzumab + chemotherapy	275/343 (80%)	[94]
			Trastuzumab + tucatinib + capecitabine	138/340 (41%)	[95]
			Trastuzumab deruxtecan	112/184 (61%)	[96]
*ERBB2* *	2	Colorectal cancer	Lapatinib + trastuzumab	9/32 (28%)	[97]
			Trastuzumab + pertuzumab	18/57 (32%)	[98]
			Trastuzumab deruxtecan	24/53 (45%)	[99]
ERBB2 *	2	Uterine serous carcinoma	Trastuzumab + carboplatin-taxol	4/9 (44%)	[100]
*ERBB2* ^¥^	2	NSCLC	Ado-trastuzumab emtansine	8/18 (44%)	[101]
			Trastuzumab deruxtecan	50/91 (55%)	[102]
*PIK3CA* ^¥^	1	Breast cancer	Alpelisib + fulvestrant	21/121 (17%	[103]
TMB-H	1	Solid tumors	Pembrolizumab	30/102 (29%)	[104]
MSI-H	1	Solid tumors	Pembrolizumab	59/149 (40%)	[105]
MSI-H	1	Colorectal cancer	Nivolumab	23/74 (31%)	[106]
			Ipilumab + nivolumab	65/119 (55%)	[107]

^¥^: mutation; *: amplification. ORR: objective response rate; NSCLC: non-small cell lung cancer; MSI-H: high microsatellite instability; TMB-H: high tumor mutational burden.

**Table 5:** Ongoing clinical trials targeting genetic alterations in GBC.

Target	Phase	Agent	Country	Trial ID
*ERBB2* signal pathway components	2	FORFIRINOX + (cetuximab, trastuzumab, gefitinib, lapatinib, everolimus, sorafenib, or crizotinib)	China	NCT03768375
*ERBB2* signal pathway components	2	GEMOX + afatinib	China	NCT04183712
*ERBB2* overexpression/ amplification	2	Trastuzumab + pertuzumab	U.S.	NCT02091141
*ERBB2* overexpression/ amplification	1,2	Tucatinib + trastuzumab + (FOLFOX or CAPOX)	U.S.	NCT04430738
*ERBB2* overexpression/ amplification or mutations	2, basket	Tucatinib + trastuzumab	U.S., Japan, Belgium	NCT04579380
*ERBB2* amplification	2	Zanidatamab	U.S., Canada, Chile, China, France, Italy, Korea, Spain, U.K.	NCT04466891
*KRAS (*or *NRAS*) mutation	1	ELI-002 immunotherapy	U.S.	NCT04853017
DNA repair gene mutations (including *ARID1A*, *ATM*, and others)	2	Olaparib	U.S.	NCT04042831
TMB ≥ 10 mutations/Mb	2	Atezolizumab	U.S.	NCT02091141

U.S.: United States; U.K.: United Kingdom; TMB: tumor mutational burden.

## Appendix A

**Table A1:** Keywords and subject headings used in the literature search.

		Database	
PubMed	EMBASE	Cochrane Reviews	Web of Science
(“Gallbladder Neoplasms”[Mesh] OR (gallbladder[tiab] AND (neoplasm * [tiab] OR cancer * [tiab] OR lesion * [tiab] OR tumor* [tiab] OR tumour * [tiab] OR carcinom * [tiab] OR malignan * [tiab]))) AND (“Mutation”[Mesh] OR “Epigenesis, Genetic”[Mesh] OR “Genome”[Mesh] OR molecular[tiab] OR gene[tiab] OR genes[tiab] OR genetic * [tiab] OR genom * [tiab] OR sequenc * [tiab] OR mutation*[tiab] OR exome * [tiab])	(‘gallbladder cancer’/exp OR (gallbladder AND (neoplasm * OR cancer* OR tumor * OR tumour * OR carcinom * OR malignan *)):ab,ti,kw) AND (‘gene mutation’/exp OR ‘gene sequence’/exp OR (exome * OR molecular OR gene OR genes OR genetic * OR genom * OR sequenc * OR mutation *):ab,ti,kw)	(gallbladder AND (neoplasm * OR cancer * OR tumor * OR tumour * OR carcinom * OR malignan *)) AND (molecular OR gene OR genes OR genetic * OR genom * OR sequenc * OR mutation * OR exome *)	(gallbladder AND (neoplasm * OR cancer * OR tumor * OR tumour * OR carcinom * OR malignan *)) AND (exome * OR molecular OR gene OR genes OR genetic * OR genom * OR sequenc * OR mutation *)

*: show keywords in a search strategy.

**Table A2:** Studies analyzing frequently mutated genes in gallbladder cancer.

Gene	WA	N	Frequency	Methods	Histology	Population	Author	Year	Ref.
*ARID1A*	14.3%	1/5	20%	WES	AC	Japan	Akita	2019	[48]
		2/14	14%	TS	N.A.	China	Li	2017	[44]
		2/47	4%	WES	AC	China	Yang	2021	[56]
		2/25	8%	targeted exome sequencing	N.A.	Korea	Chae	2019	[79]
		3/24	13%	NGS	N.A.	America	Okamura	2021	[27]
		3/26	12%	NGS	N.A.	Italy	Simbolo	2014	[61]
		4/54	7%	NGS	AC	Greece	Papadopoulou	2018	[40]
		4/39	10%	WES/WGS	N.A.	Japan	Ebata	2021	[57]
		15/144	10%	WES	AC	India, Korea, Chile	Pandey	2020	[46]
		7/58	12%	ultra-deep targeted NGS	AC	China	Lin	2019	[70]
		8/32	25%	targeted NGS	AC	Chile, Japan	Narayan	2019	[73]
		10/55	18%	NGS	N.A.	America	Javle	2016	[23]
		123/760	16%	NGS	N.A.	America	Abdel-Wahab	2020	[19]
*ARID2*	8.8%	1/32	3%	targeted NGS	AC	Chile, Japan	Narayan	2019	[73]
		1/17	6%	WES	N.A.	India	Iyer	2019	[29]
		1/14	7%	TS	N.A.	China	Li	2017	[44]
		1/24	4%	NGS	N.A.	America	Okamura	2021	[27]
		3/25	12%	targeted exome sequencing	N.A.	Korea	Chae	2019	[79]
		3/54	6%	NGS	AC	Greece	Papadopoulou	2018	[40]
		3/39	8%	WES/WGS	N.A.	Japan	Ebata	2021	[57]
		19/144	13%	WES	AC	India, Korea, Chile	Pandey	2020	[46]
		2/12	17%	NGS	N.A.	China	Li	2020	[47]
		7/58	12%	ultra-deep targeted NGS	AC	China	Lin	2019	[70]
		8/47	17%	WES	AC	China	Yang	2021	[56]
		61/760	8%	NGS	N.A.	America	Abdel-Wahab	2020	[19]
*ATM*	6.3%	0/4	0%	TS	AC	Korea	Yoo	2016	[37]
		1/14	7%	NGS	N.A.	Japan	Noguchi	2017	[32]
		1/58	2%	NGS	AC	America	Maynard	2020	[41]
		2/25	8%	targeted exome sequencing	N.A.	Korea	Chae	2019	[79]
		5/32	16%	targeted NGS	AC	Chile, Japan	Narayan	2019	[73]
		3/17	18%	WES	N.A.	India	Iyer	2019	[29]
		4/58	7%	ultra-deep targeted NGS	AC	China	Lin	2019	[70]
		45/760	6%	NGS	N.A.	America	Abdel-Wahab	2020	[19]
*CDKN2A*	8.5%	0/24	0%	NGS	N.A.	America	Okamura	2021	[27]
		0/5	0%	WES	AC	Japan	Akita	2019	[48]
		0/4	0%	TS	AC	Korea	Yoo	2016	[37]
		1/11	9%	ultra-deep targeted NGS	N.A.	India	Yadav	2017	[77]
		1/26	4%	NGS	N.A.	Italy	Simbolo	2014	[61]
		1/14	7%	NGS	N.A.	Japan	Noguchi	2017	[32]
		13/144	9%	WES	AC	India, Korea, Chile	Pandey	2020	[46]
		1/12	8%	NGS	N.A.	China	Li	2020	[47]
		2/25	8%	targeted exome sequencing	N.A.	Korea	Chae	2019	[79]
		2/32	6%	targeted NGS	AC	Chile, Japan	Narayan	2019	[73]
		2/14	14%	TS	N.A.	China	Li	2017	[44]
		4/13	31%	PCR-SSCP + DS	N.A.	Korea	Kim	2001	[31]
		6/58	10%	ultra-deep targeted NGS	AC	China	Lin	2019	[70]
		64/760	8%	NGS	N.A.	America	Abdel-Wahab	2020	[19]
*CTNNB1*	6.4%	0/21	0%	SNaPshot	AC	America	Moy	2015	[53]
		0/68	0%	WES + Sanger seq	AC	Japan	Akita	2019	[48]
		0/26	0%	NGS	N.A.	Italy	Simbolo	2014	[61]
		0/4	0%	TS	AC	Korea	Yoo	2016	[37]
		0/14	0%	NGS	N.A.	Japan	Noguchi	2017	[32]
		0/58	0%	ultra-deep targeted NGS	AC	China	Lin	2019	[70]
		1/25	4%	SNaPshot	N.A.	America	Borger	2012	[30]
		2/46	4%	mass array + seq	AC	India	Kumari	2014	[68]
		2/14	14%	TS	N.A.	China	Li	2017	[44]
		2/17	12%	WES	N.A.	India	Iyer	2019	[29]
		18/144	13%	WES	AC	India, Korea, Chile	Pandey	2020	[46]
		9/50	18%	PCR-SSCP + seq	AC	India	Dixit	2020	[36]
		19/47	40%	NGS	AC	Greece	Papadopoulou	2018	[40]
		30/760	4%	NGS	N.A.	America	Abdel-Wahab	2020	[19]
*ELF3*	18.6%	29/144	20%	WES	AC	India, Korea, Chile	Pandey	2020	[46]
		5/39	13%	WES/WGS	N.A.	Japan	Ebata	2021	[57]
*ERBB2*	6.7%	0/21	0%	SNaPshot	AC	America	Moy	2015	[53]
		0/46	0%	mass array	AC	India	Kumari	2014	[68]
		0/4	0%	TS	AC	Korea	Yoo	2016	[37]
		0/58	0%	ultra-deep targeted NGS	AC	China	Lin	2019	[70]
		1/26	4%	NGS	N.A.	Italy	Simbolo	2014	[61]
		2/32	6%	targeted NGS	AC	Chile, Japan	Narayan	2019	[73]
		1/14	7%	TS	N.A.	China	Li	2017	[44]
		2/54	4%	NGS	AC	Greece	Papadopoulou	2018	[40]
		2/5	40%	WES	AC	Japan	Akita	2019	[48]
		2/25	8%	targeted exome sequencing	N.A.	Korea	Chae	2019	[79]
		3/50	6%	NGS	N.A.	India	Patel	2020	[39]
		3/17	18%	WES	N.A.	India	Iyer	2019	[29]
		3/24	13%	NGS	N.A.	America	Okamura	2021	[27]
		3/39	8%	WES/WGS	N.A.	Japan	Ebata	2021	[57]
		4/47	9%	WES	AC	China	Yang	2021	[56]
		22/144	15%	WES	AC	India, Korea, Chile	Pandey	2020	[46]
		6/111	5%	NGS	N.A.	America	Mondaca	2019	[38]
		5/12	42%	NGS	N.A.	China	Li	2020	[47]
		11/157	7%	WES	N.A.	China	Li	2019	[18]
		40/760	5%	NGS	N.A.	America	Abdel-Wahab	2020	[19]
*ERBB3*	5.4%	2/5	40%	WES	AC	Japan	Akita	2019	[48]
		1/11	9%	ultra-deep targeted NGS	N.A.	India	Yadav	2017	[77]
		1/17	6%	WES	N.A.	India	Iyer	2019	[29]
		0/24	0%	NGS	N.A.	America	Okamura	2021	[27]
		0/32	0%	targeted NGS	AC	Chile, Japan	Narayan	2019	[73]
		3/39	8%	WES/WGS	N.A.	Japan	Ebata	2021	[57]
		4/47	9%	WES	AC	China	Yang	2021	[56]
		3/50	6%	NGS	N.A.	India	Patel	2020	[39]
		3/54	6%	NGS	AC	Greece	Papadopoulou	2018	[40]
		0/58	0%	ultra-deep targeted NGS	AC	China	Lin	2019	[70]
		17/144	12%	WES	AC	India, Korea, Chile	Pandey	2020	[46]
		12/157	8%	WES	N.A.	China	Li	2019	[18]
		30/760	4%	NGS	N.A.	America	Abdel-Wahab	2020	[19]
*KMT2D*	7.7%	0/58	0%	ultra-deep targeted NGS	AC	China	Lin	2019	[70]
		1/17	6%	WES	N.A.	India	Iyer	2019	[29]
		1/14	7%	TS	N.A.	China	Li	2017	[44]
		5/32	16%	targeted NGS	AC	Chile, Japan	Narayan	2019	[73]
		5/54	9%	NGS	AC	Greece	Papadopoulou	2018	[40]
		5/47	11%	WES	AC	China	Yang	2021	[56]
*KRAS*	10.3%	1/42	2%	nested PCR, PCR-RFLP + DS	AC	Japan, Hungary	Nagahashi	2008	[35]
		0/27	0%	Oncomap	AC	America	Deshpande	2011	[66]
		0/29	0%	nested PCR, PCR-RFLP + DS	AC	Peru	Vidaurre	2019	[82]
		1/35	3%	PCR + seq	AC	Bolivia	Asai	2014	[42]
		1/25	4%	SNaPshot	N.A.	America	Borger	2012	[30]
		1/46	2%	mass array+ seq	AC	India	Kumari	2014	[68]
		1/4	25%	TS	AC	Korea	Yoo	2016	[37]
		1/24	4%	NGS	N.A.	America	Okamura	2021	[27]
		1/14	7%	TS	N.A.	China	Li	2017	[44]
		2/9	22%	PCR + seq	AC	Japan	Shibata	2008	[33]
		2/64	3%	Seq	AC	China	Rashid	2002	[49]
		2/29	7%	PCR	AC	America	Pai	2011	[62]
		2/60	3%	PCR + DS	AC	Taiwan	Huang	2017	[67]
		2/14	14%	NGS	N.A.	Japan	Noguchi	2017	[32]
		2/21	10%	SNaPshot	AC	America	Moy	2015	[53]
		2/12	17%	NGS	N.A.	China	Li	2020	[47]
		2/32	6%	targeted NGS	AC	Chile, Japan	Narayan	2019	[73]
		3/15	20%	PCR-RFLP + DS	N.A.	Korea	Kim	2001	[31]
		4/35	11%	DS	N.A.	Taiwan	Chang	2013	[25]
		4/34	12%	PCR	N.A.	Korea	Kim	2015	[60]
		4/68	6%	WES and Sanger seq	AC	Japan	Akita	2019	[48]
		6/144	4%	WES	AC	India, Korea, Chile	Pandey	2020	[46]
		4/58	7%	ultra-deep targeted NGS	AC	China	Lin	2019	[70]
		5/55	9%	NGS	N.A.	America	Javle	2016	[23]
		5/26	19%	NGS	N.A.	Italy	Simbolo	2014	[61]
		5/25	20%	targeted exome sequencing	N.A.	Korea	Chae	2019	[79]
		5/157	3%	WES	N.A.	China	Li	2019	[18]
		6/54	11%	NGS	AC	Greece	Papadopoulou	2018	[40]
		8/21	38%	PCR-RFLP	AC	India	Singh	2004	[63]
		8/34	24%	Seq	AC	India	Sharma	2017	[65]
		9/81	11%	PCR	N.A.	Japan	Tomioka	2019	[45]
		10/17	59%	PCR	N.A.	Japan	Nagai	2002	[24]
		10/20	50%	PCR + RFLP and DS	N.A.	Korea	Kim	2000	[50]
		12/25	48%	PCR-RFLP	AC	India	Shukla	2020	[34]
		16/39	41%	PCR-RFLP	N.A.	India	Kazmi	2013	[71]
		72/760	9%	NGS	N.A.	America	Abdel-Wahab	2020	[19]
*PIK3CA*	10.0%	0/34	0%	PCR	N.A.	Korea	Kim	2015	[60]
		0/58	0%	ultra-deep targeted NGS	AC	China	Lin	2019	[70]
		1/23	4%	PCR+ seq	N.A.	Switzerland	Riener	2008	[72]
		1/68	1%	WES + Sanger seq	AC	Japan	Akita	2019	[48]
		1/14	7%	TS	N.A.	China	Li	2017	[44]
		1/25	4%	targeted exome sequencing	N.A.	Korea	Chae	2019	[79]
		2/46	4%	mass array + seq	AC	India	Kumari	2014	[68]
		2/4	50%	TS	AC	Korea	Yoo	2016	[37]
		2/21	10%	SNaPshot	AC	America	Moy	2015	[53]
		2/24	8%	NGS	N.A.	America	Okamura	2021	[27]
		2/26	8%	NGS	N.A	Italy	Simbolo	2014	[61]
		3/27	11%	Oncomap	AC	America	Deshpande	2011	[66]
		3/25	12%	SNaPshot	N.A.	America	Borger	2012	[30]
		3/14	21%	NGS	N.A.	Japan	Noguchi	2017	[32]
		3/21	14%	targeted NGS	AC	Chile, Japan	Narayan	2019	[73]
		11/144	8%	WES	AC	India, Korea, Chile	Pandey	2020	[46]
		2/12	17%	NGS	N.A.	China	Li	2020	[47]
		5/47	11%	WES	AC	China	Yang	2021	[56]
		6/157	4%	WES	N.A.	China	Li	2019	[18]
		7/55	13%	NGS	N.A.	America	Javle	2016	[23]
		7/34	21%	Seq	AC	India	Sharma	2017	[65]
		8/130	6%	TS	N.A.	China	Zhao	2016	[78]
		10/54	19%	NGS	AC	Greece	Papadopoulou	2018	[40]
		102/760	13%	NGS	N.A.	America	Abdel-Wahab	2020	[19]
*SHH*	20.0%	10/50	20%	PCR + SSCP + seq	AC	India	Dixit	2017	[26]
*SMAD4*	13.1%	0/4	0%	TS	AC	Korea	Yoo	2016	[37]
		1/17	6%	WES	N.A.	India	Iyer	2019	[29]
		1/5	20%	WES	AC	Japan	Akita	2019	[48]
		2/12	17%	NGS	N.A.	China	Li	2020	[47]
		2/26	8%	NGS	N.A.	Italy	Simbolo	2014	[61]
		2/14	14%	NGS	N.A.	Japan	Noguchi	2017	[32]
		3/24	13%	NGS	N.A.	America	Okamura	2021	[27]
		11/144	8%	WES	AC	India, Korea, Chile	Pandey	2020	[46]
		3/11	27%	ultra-deep targeted NGS	N.A.	India	Yadav	2017	[77]
		4/25	16%	targeted exome sequencing	N.A.	Korea	Chae	2019	[79]
		10/32	33%	targeted NGS	AC	Chile, Japan	Narayan	2019	[73]
		9/55	16%	NGS	N.A.	America	Javle	2016	[23]
		16/58	28%	ultra-deep targeted NGS	AC	China	Lin	2019	[70]
		92/760	12%	NGS	N.A.	America	Abdel-Wahab	2020	[19]
*SRCAP*	13.0%	7/54	13%	NGS	AC	Greece	Papadopoulou	2018	[40]
*STK11*	7.2%	0/68	0%	WES and Sanger seq	AC	Japan	Akita	2019	[48]
		1/24	4%	NGS	N.A.	America	Okamura	2021	[27]
		1/26	4%	NGS	N.A.	Italy	Simbolo	2014	[61]
		10/144	7%	WES	AC	India, Korea, Chile	Pandey	2020	[46]
		3/39	8%	WES/WGS	N.A.	Japan	Ebata	2021	[57]
		4/58	7%	ultra-deep targeted NGS	AC	China	Lin	2019	[70]
		64/760	8%	NGS	N.A.	America	Abdel-Wahab	2020	[19]
*SYNE1*	6.2%	3/54	6%	NGS	AC	Greece	Papadopoulou	2018	[40]
		1/11	9%	ultra-deep targetedNGS	N.A.	India	Yadav	2017	[77]
*TP53*	57.0%	1/21	5%	SNaPshot	AC	America	Moy	2015	[53]
		1/25	4%	SNaPshot	N.A.	America	Borger	2012	[30]
		2/4	50%	TS	AC	Korea	Yoo	2016	[37]
		2/11	18%	ultra-deep targeted NGS	N.A.	India	Yadav	2017	[77]
		3/5	60%	WES	AC	Japan	Akita	2019	[48]
		4/46	9%	mass array + seq	AC	India	Kumari	2014	[34]
		5/14	36%	PCR-SSCP + DS	N.A.	Korea	Kim	2001	[31]
		6/17	36%	PCR-SSCP + seq	N.A.	Japan	Nagai	2002	[24]
		6/17	36%	WES	N.A.	India	Iyer	2019	[29]
		7/14	50%	TS	N.A.	China	Li	2017	[44]
		7/29	24%	nested PCR, PCR-RFLP + DS	AC	Peru	Vidaurre	2019	[82]
		8/12	67%	NGS	N.A.	China	Li	2020	[47]
		9/14	64%	NGS	N.A.	Japan	Noguchi	2017	[32]
		17/40	43%	nested PCR +DS	AC	Japan, Hungary	Nagahashi	2008	[35]
		11/25	44%	PCR-SSCP	AC	India	Shukla	2020	[34]
		11/24	46%	NGS	N.A.	America	Okamura	2021	[27]
		12/26	46%	NGS	N.A.	Italy	Simbolo	2014	[61]
		1535	43%	PCR + seq	AC	Bolivia	Asai	2014	[42]
		16/25	64%	targeted exome sequencing	N.A.	Korea	Chae	2019	[79]
		22/32	69%	targeted NGS	AC	Chile, Japan	Narayan	2019	[73]
		18/59	31%	Seq	AC	Austria	Puhalla	2004	[69]
		72/144	50%	WES	AC	India, Korea, Chile	Pandey	2020	[46]
		19/39	49%	WES/WGS	N.A.	Japan	Ebata	2021	[57]
		20/30	67%	PCR + DS	AC	Chile	Moreno	2005	[81]
		23/54	43%	NGS	AC	Greece	Papadopoulou	2018	[40]
		32/55	58%	NGS	N.A.	America	Javle	2016	[23]
		42/58	72%	ultra-deep targeted NGS	AC	China	Lin	2019	[70]
		541/760	71%	NGS	N.A.	America	Abdel-Wahab	2020	[19]

WA: weighted average (calculated by using the number of samples analyzed in a study as the weight); N.A.: not available; AC: adenocarcinoma; NGS: next-generation sequencing; PCR: polymerase chain reaction; Seq: sequencing; DS: direct sequencing; WES: whole-exome sequencing; WGS: whole-genome sequencing; SSCP: single-strand conformation polymorphism; RFLP: restriction fragment length polymorphism; TS: targeted sequencing.
